# Neuroendocrine neoplasms in uncommon gastrointestinal sites: A case series and literature review

**DOI:** 10.4102/sajr.v30i1.3455

**Published:** 2026-06-30

**Authors:** Neha Chhabra, Angie Garg

**Affiliations:** 1 Department of Radiodiagnosis and Interventional Radiology, Homi Bhabha Cancer Hospital and Research Centre, Homi Bhabha National Institute, Medicity, New Chandigarh, Punjab, India

**Keywords:** neuroendocrine neoplasm, neuroendocrine tumour, duodenum, gallbladder, rectum, somatostatin receptor imaging

## Abstract

Neuroendocrine neoplasms (NENs) are rare, heterogeneous tumours arising from neuroendocrine cells located throughout the body. The gastrointestinal (GI) tract is the most frequent site of origin, but lesions in unusual locations such as the duodenum, gallbladder and rectum are diagnostically challenging because of their atypical presentation and radiological appearance. This series describes three patients with NENs at uncommon GI sites, highlighting imaging findings, histopathological features and management tailored to the tumour site and grade.

**Contribution:**

Although rare, NENs at uncommon GI sites should be included in the differential diagnosis of hypervascular lesions. Accurate diagnosis requires integration of cross-sectional imaging, somatostatin-receptor PET and immunohistochemistry, guiding individualised therapy.

## Introduction

Neuroendocrine neoplasms (NENs) are uncommon malignancies that originate from neuroendocrine cells distributed in multiple organs. They represent approximately 0.5% of all cancers and exhibit variable biological behaviours.^1^ The gastrointestinal (GI) and bronchopulmonary tracts are the predominant primary sites.^2^

While lesions in the ileum, jejunum and pancreas are well-documented, NENs arising from the duodenum, gallbladder and rectum are infrequently encountered and may mimic adenocarcinoma, both radiologically and clinically. Early diagnosis is crucial for optimal management and prognosis. This case series presents three patients with NENs in these unusual GI locations, emphasising imaging characteristics, histopathology and treatment outcomes.

## Ethical considerations

This study was in accordance with the ethical standards of the 1964 Helsinki Declaration and its later amendments or comparable ethical standards. Written informed consent was obtained from all individual participants involved in the study.

## Case presentations

### Case 1: Duodenal neuroendocrine tumour with solitary liver metastasis

A 65-year-old man presented with abdominal pain, dyspepsia, weight loss and anorexia. Routine biochemistry and tumour markers [CA 19-9, carcinoembryonic antigen (CEA), and alphafetoprotein (AFP)] were normal. Ultrasound revealed a solitary hepatic lesion in segment VI. Triphasic CT demonstrated an arterially enhancing hepatic lesion with portal-phase washout and a hypervascular polypoidal mass in the second part of the duodenum (Figure 1a).

**FIGURE 1:**
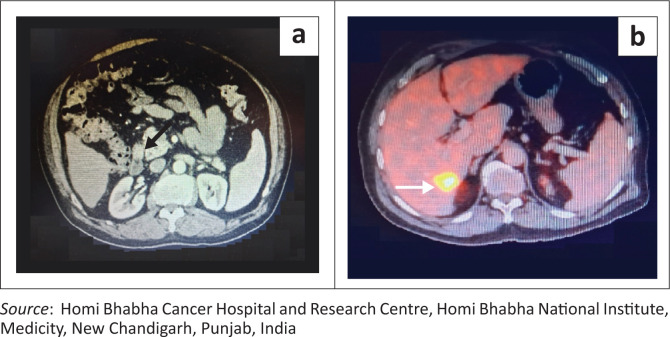
CT of the upper abdomen reveals a mildly hypervascular polypoidal lesion in the second part of duodenum (black arrow) in (a) and an FDG avid metastatic lesion in the right lobe of the liver (white arrow) in (b).

FDG-PET revealed uptake in the liver lesion (SUV 8.3, Figure 1b), but not in the duodenal mass. Endoscopic resection of the 1.8 cm polypoid lesion revealed nests of small round cells with granular cytoplasm; immunohistochemistry (IHC) was positive for PANCK and synaptophysin, negative for chromogranin A and Ki-67 = 1%, diagnosed as well-differentiated NET Grade 1. Fine needle aspiration cytology (FNAC) of the hepatic lesion confirmed metastasis. The patient underwent microwave ablation of the hepatic lesion and was treated with octreotide therapy. Except for a few episodes of diarrhoea, nausea and stomach pain at the initiation of therapy, the patient is doing well with no recurrence to date.

### Case 2: Gallbladder neuroendocrine tumour with hepatic infiltration

A 45-year-old woman presented with nausea, upper-abdominal pain and marked weight loss. Tumour markers (AFP, CEA, CA 19-9) were normal; serum chromogranin A was elevated at 2305 ng/mL. Contrast-enhanced CT abdomen showed a large, arterially enhancing mass replacing the gallbladder and infiltrating the adjacent IV, V, VI and VIII hepatic segments (Figure 2).

**FIGURE 2:**
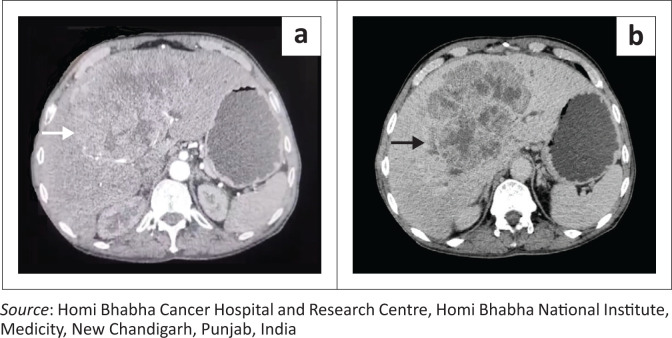
CECT abdomen demonstrating a hypervascular gallbladder-replacing mass (white arrow) on arterial phase imaging (a), infiltrating the adjacent liver parenchyma and revealing washout on the portal venous phase (b).

Ultrasound-guided biopsy revealed monotonous tumour cells with granular chromatin (mitotic rate 8–9/10 HPF). Immunohistochemistry was positive for synaptophysin, chromogranin, AE1/AE3; Ki-67 = 25% – 30%, diagnosed as well-differentiated NET Grade 3. DOTA-PET/CT demonstrated intense tracer uptake (SUV 52). As a result of encasement of the right hepatic artery and extensive hepatic infiltration, surgery was deferred; the patient was started on capecitabine and temozolomide. Unfortunately, the patient developed severe myelosuppression and demised due to respiratory infection.

### Case 3: Rectal neuroendocrine carcinoma with peritoneal metastases

A 53-year-old man presented with intestinal obstruction. Digital rectal examination revealed a circumferential constricting growth. MRI pelvis demonstrated mural thickening of the mid- and lower-rectum with anal canal involvement (Figure 3).

**FIGURE 3:**
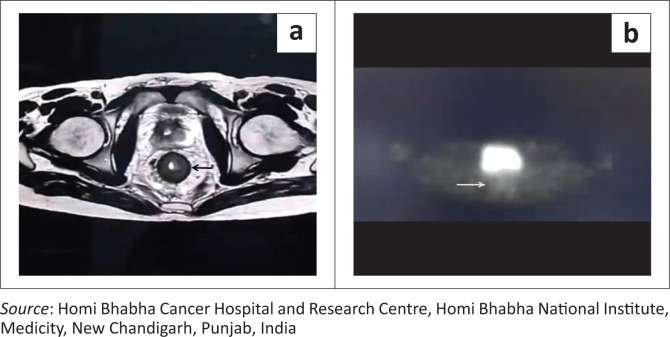
MRI pelvis (a) revealing circumferential rectal wall thickening (black arrow). The corresponding HYNIC-TOC SPECT/CT (b) shows absence of tracer uptake (white arrow) in the rectum behind the urinary bladder.

Biopsy showed cords of tumour cells with granular chromatin; IHC was positive for synaptophysin, chromogranin, INSM1, AE1/AE3; Ki-67 ≈ 30%, diagnosed as neuroendocrine carcinoma (NEC) Grade 3. CT revealed mesocolonic deposits; laparotomy PCI = 12/39. HYNIC-TOC SPECT/CT showed no significant SSTR expression; the patient was started on etoposide–cisplatin chemotherapy. The patient continued chemotherapy for 5 months; he subsequently demised from renal failure as a result of drug toxicity.

## Discussion

Neuroendocrine neoplasms constitute a heterogeneous group of epithelial tumours arising from neuroendocrine cells dispersed throughout the body. Although their overall incidence is low, the number of detected cases has steadily increased over recent decades due to improved imaging modalities and heightened clinical awareness.^1,2^ Most NENs occur in the GI tract (approximately two-thirds) and bronchopulmonary system. Primary lesions in the duodenum, gallbladder or rectal region are less frequent, but clinically significant because of their aggressive course and diagnostic ambiguity.^3^

Duodenal NENs are rare, accounting for only 2% – 3% of all GI NENs and about 1% – 3% of duodenal tumours.^4^ They originate from enterochromaffin cells located in the submucosa of the duodenum and are most often found in its first and second parts. These tumours are typically small, solitary and well-differentiated, and are frequently detected incidentally during endoscopic evaluations performed for unrelated indications.^5^ They need to be differentiated from adenocarcinomas which are hypovascular, large, infiltrative and ulcerated masses showing desmoplasia. Metastases from duodenal adenocarcinomas are hypovascular, whereas those from NETs are hypervascular. Metastatic spread occurs primarily to regional lymph nodes (40% – 60%) and, less frequently, to the liver (10%).^6^ The majority of duodenal NENs are non-functional; however, a minority secrete biologically active peptides such as gastrin, somatostatin or serotonin, resulting in distinct clinical syndromes like Zollinger–Ellison or carcinoid syndrome.^7^

Contrast-enhanced CT and MRI characteristically demonstrate hyperenhancing submucosal nodules during the arterial phase. Endoscopic ultrasonography further aids in assessing the depth of invasion and nodal involvement.^8^ Somatostatin receptor scintigraphy or 68Ga-DOTATATE PET/CT provides high sensitivity in detecting small lesions and metastatic disease.^9^ In Case 1, imaging and histopathology confirmed a well-differentiated Grade 1 duodenal neuroendocrine tumour (NET) with a solitary hepatic metastasis, which was successfully managed with endoscopic resection, local ablation and octreotide therapy.

Primary gallbladder NENs (GB-NENs) are extremely uncommon, representing approximately 0.5% of all NETs and 2% of gallbladder malignancies.^10^ Because the normal gallbladder mucosa lacks neuroendocrine cells, their origin is thought to be secondary to intestinal or gastric metaplasia induced by chronic inflammation, particularly due to cholelithiasis or cholecystitis.^11^ These tumours are typically aggressive and often diagnosed at an advanced stage owing to non-specific clinical manifestations such as abdominal pain, anorexia or weight loss.^12^

Radiologically, GB-NENs can manifest as polypoidal intraluminal lesions, irregular mural thickening or gallbladder-replacing masses with well-defined rounded margins and intact enhancing mucosa. The ‘gallbladder-replacing’ pattern has been reported as the most frequent presentation as seen in Case 2.^13^ On CT, late arterial phase hyperenhancement of the tumour, along with hypervascular, large, rapidly growing, necrotic nodal and hepatic metastases, favours a neuroendocrine origin over conventional adenocarcinoma.^14^ MRI typically demonstrates a T1 hypointense and T2 hyperintense lesion with strong gadolinium enhancement. Nuclear imaging with 68Ga-DOTATATE or 18F-FDG PET/CT provides complementary information on receptor status and tumour grade.^15^ In contrast, GB adenocarcinomas present as hypovascular masses with an irregular outline and an ulcerated mucosa. Surgical resection was precluded in the presented case due to vascular invasion of the right hepatic artery and extensive hepatic involvement; the patient was treated with capecitabine and temozolomide chemotherapy.

Rectal NENs account for approximately 20% of all GI NENs and are second in frequency only to small intestinal NENs.^16^ Their incidence has increased markedly with widespread adoption of screening colonoscopy and high-resolution endoscopic imaging. Most rectal NENs arise from L-cells in the submucosa and are non-functional; therefore, carcinoid syndrome is rare.^17^ The clinical presentation depends on tumour size and depth. Small lesions (< 1 cm) are often asymptomatic, while larger or poorly differentiated lesions may cause pain, bleeding or obstruction. On MRI, rectal NENs typically appear as submucosal masses with intermediate T1 and mildly hyperintense T2 signal, demonstrating avid enhancement on post-contrast sequences.^18^ Rectal NENs typically appear as small, solitary, submucosal nodules showing intense homogenous enhancement with smaller lymph nodes, whereas rectal adenocarcinomas present as large, ulcerated and irregular masses causing mucosal destruction and penetrating deeper into the muscularis with larger size nodes.

Prognosis correlates with tumour grade and size. Well-differentiated, localised tumours have excellent outcomes following endoscopic or surgical excision, whereas poorly differentiated NECs are aggressive, with a high propensity for nodal and distant metastases.^19^ Case 3 demonstrated a Grade 3 rectal NEC with peritoneal deposits and high Ki-67 proliferation (30%), requiring platinum-based chemotherapy. A lack of somatostatin receptor expression on HYNIC-TOC SPECT/CT excluded the option of peptide receptor radionuclide therapy (PRRT).

The 2022 WHO classification divides NENs into well-differentiated NETs and poorly differentiated NECs, based on differentiation, morphology and genetic alterations. Well-differentiated NETs retains organoid architecture with uniform cytology, whereas poorly differentiated NECs have sheets of small or large cells with high atypia and necrosis. Well-differentiated NETs are further classified into G1, G2, G3 depending on Ki 67 index < 3, 3–20, > 20 (20% – 55%) and mitotic activity < 2, 2–20, > 20, whereas NEC or poorly differentiated types have Ki 67 > 20, usually > 70% – 80%. Neuroendocrine tumours have MEN 1, DAXX and ATRX mutations with strong or diffuse SSTR expression, and treatment includes somatostatin analogues, PRRT, capecitabine and temozolomide. Neuroendocrine tumours have a good prognosis. Neuroendocrine carcinomas are associated with TP53 and RB 1 mutations with low or absent SSTR expression and are managed with platinum-based therapy consisting of cisplatin and etoposide. They have a poor prognosis.^20^ Mixed neuroendocrine-non-neuroendocrine neoplasms (MiNENs) consist of either NET or NEC and a non-neuroendocrine component like adenocarcinoma, with each component > 30%.^21,22^

Functional imaging in NETs is performed with dual tracer PET/CT using somatostatin receptor (68Ga-DOTATATE or 68Ga-DOTATOC) and 18F-FDG. Combining 68GaDOTATATE and F18-FDG has many benefits. Firstly, it helps decipher tumour heterogeneity. Neuroendocrine tumours can have variable mixed behaviour in the same patient as seen in Case 1 where the liver lesion was FDG avid, but not the duodenal lesion. Secondly, it helps in prognostic assessment by determining the NETPET grade. A scoring system from 0 to 5 has been assigned comparing the two radiotracer uptake patterns:

- P0 – No uptake, normal scan on both.
- P1 – purely SSTR avid (good prognosis).
- P2-4 – Intermediate pattern.
- P5 – FDG avid, SSTR negative (poor prognosis).

NETPET scores correlate with overall survival and progression free survival.^23,24^

Key prognostic markers in NETs are – Ki67, Chromogranin A, Synaptophysin and 5-HIAA. Emerging specific markers are NETest, genetic mutations like MEN 1, and novel pulmonary markers are OTP and CD1a. Ki67 is the most reliable marker for tumour grading. Chromogranin A is a general marker which can also be falsely elevated in renal failure and with proton pump inhibitor use. Synaptophysin is a highly specific IHC marker for neuroendocrine differentiation. 5-HIAA is present in serotonin producing carcinoid tumours. Neuroendocrine tumour test is a molecular and/or genetic signature analysis that offers high sensitivity for disease detection, progression and monitoring. Insulinoma associated protein 1 (INSM1) is a newer, reliable marker for neuroendocrine differentiation that stains the nucleus.^25^

Management of NENs requires a multidisciplinary approach integrating surgery, medical therapy and targeted molecular imaging. For localised disease, surgical resection remains the mainstay of curative treatment. In metastatic or unresectable cases, medical therapy with somatostatin analogues (SSAs) such as octreotide LAR or lanreotide, provides symptomatic relief and exerts antiproliferative effects, as demonstrated in the PROMID trial.^26^ Other systemic options include interferon-α for tumour stabilisation; mTOR inhibitors (everolimus) and tyrosine kinase inhibitors (sunitinib) for advanced disease; PRRT with 177Lu-DOTATATE for somatostatin receptor-positive lesions; and capecitabine and temozolomide for advanced metastatic high-grade NETs.^27^ High-grade NECs, owing to their aggressive biology, are treated with platinum-based systemic chemotherapy – typically a combination of etoposide and cisplatin – mirroring protocols for small-cell carcinoma.^28^

The prognosis of NENs varies significantly by site and grade. Well-differentiated NETs exhibit indolent behaviour with 5-year survival rates exceeding 80%, whereas poorly differentiated NECs carry a median survival of less than 12 months despite aggressive therapy.^29^ Early recognition of atypical GI sites and appropriate receptor imaging are therefore vital for improving outcomes.

## Conclusion

Neuroendocrine neoplasms at uncommon GI sites are diagnostically challenging, but must be considered when hypervascular lesions are encountered. A multidisciplinary approach combining triphasic CT, MRI and functional imaging (PET/CT) with immunocytochemistry ensures accurate diagnosis and guides individualised management based on tumour grade and receptor expression. The role of dual tracer approach to decipher the tumor heterogeneoty and the inclusion of the NETPET scoring system (P0–P5) for prognostic assessment based on tracer uptake patterns has been emphasized in this case series.
